# Transcriptomic microRNA Profiling of Dendritic Cells in Response to Gut Microbiota-Secreted Vesicles

**DOI:** 10.3390/cells9061534

**Published:** 2020-06-23

**Authors:** Natalia Díaz-Garrido, Sarah Bonnin, Marta Riera, Rosa Gíménez, Josefa Badia, Laura Baldomà

**Affiliations:** 1 Secció de Bioquímica i Biología Molecular, Departament de Bioquímica i Fisiologia, Facultat de Farmàcia i Ciències de l’Alimentació, Universitat de Barcelona, 08028 Barcelona, Spain; ndiazgarrido@ub.edu (N.D.-G.); 14mriera@gmail.com (M.R.); rgimenez@ub.edu (R.G.); josefabadia@ub.edu (J.B.); 2 Institut de Biomedicina de la Universitat de Barcelona (IBUB), Institut de Recerca Sant Joan de Déu (IRSJD), 08950 Barcelona, Spain; 3 Bioinformatics core facility, Centre for Genomic Regulation (CRG), The Barcelona Institute of Science and Technology, Dr. Aiguader 88, 08003 Barcelona, Spain; sarah.bonnin@crg.eu

**Keywords:** microbiota–host communication, immune modulation, intestinal homeostasis, miRNAs, gene expression, bacterial extracellular vesicles, probiotics, *E. coli* Nissle 1917

## Abstract

The interconnection between nutrients, metabolites and microbes is a key factor governing the healthy/pathological status of an individual. Thus, microbiota-based research is essential in order to better understand human health and nutrition. Gut bacteria release membrane vesicles (MVs) as an intercellular communication mechanism that allows the direct delivery of factors that prime the host’s innate immune system. We have previously shown that MVs from intestinal *E. coli* activate dendritic cells (DCs) in a strain-specific manner. To gain insights into the regulatory mechanisms involved, here, we have used an RNA deep sequencing approach to identify differentially expressed miRNAs (microRNAs) in DCs which are challenged by the MVs of the probiotic Nissle 1917 (EcN) or the commensal ECOR12. MicroRNAs are post-transcriptional regulatory mediators that permit the fine tuning of signaling pathways. This approach allowed the identification of a common set of miRNAs which are modulated by MVs from both strains and miRNAs which are differentially expressed in response to EcN or ECOR12 MVs. Based on the differential expression of the target genes and subsequent validation experiments, we correlated some of the selected miRNAs with the reported cytokine profile and specific T cell responses. As far as we know, this is the first study to analyze the regulation of miRNAs in DCs by MVs released by gut microbiota.

## 1. Introduction

The human intestine holds trillions of microbes that live in a symbiotic relationship with the host. This diverse, complex microbial community, known as the gut microbiota, is considered a hidden organ that performs an essential role in maintaining homeostasis and human health. In fact, the gut microbiota exerts a wide range of effects on the intestinal mucosa [1]. Besides its contribution to food digestion and nutrient metabolism, the gut microbiota is essential for the host’s immune system development and for the modulation of the gut barrier and immune responses. Nowadays, microbiome research is essential to better understand human health, immunity and nutrition. The interconnection between nutrients, metabolites and microbes is a key factor governing the healthy/pathological status of an individual. The high plasticity of the human microbiome sets the basis for new therapeutic strategies aimed at restoring the altered gut microbiota’s balance. The administration of prebiotics or probiotics are among these interventions, which basically try to exploit the beneficial effects of the commensal microbiota [2]. 

The gut microbiota establishes dynamic and reciprocal interactions with the intestinal epithelium and the immune system. The detection of microbes by epithelial and gut-associated innate immune cells is mediated by pattern recognition receptors (PRRs) that specifically recognize conserved microbial-associated molecular patterns (MAMPs). The interactions of PRRs with their specific ligands activates signaling pathways that result in the secretion of chemokines, cytokines and antimicrobial peptides that help to control the gut’s microbial population. This feedback control is crucial in restricting immune activation and preserving mutualistic associations between the microbiota and the host. Immune homeostasis depends on the ability of intestinal cells to distinguish between pathogens and commensals. In addition to common MAMPs, pathogens express virulence factors that enable bacteria to infect host epithelial cells and redirect signal transduction pathways, shifting the outcomes of immune responses. The host’s response to pathogens leads to controlled inflammation that assists pathogen eradication. The response to symbiotic microbiota is known as tolerance, a state that depends on highly regulated innate and adaptive immune responses that contribute to basal immune training [3].

The sampling of gut microbes is mainly mediated by cells of the mucosal innate immune system, which is comprised of resident macrophages and dendritic cells (DCs). DCs are central players in the immune system. These antigen-presenting cells can receive information from microbe-activated epithelial cells or connect the luminal environment by extending their dendrites through the inner mucosal lining. By this last mechanism, DCs directly sample gut microbes and orchestrate appropriate immune responses. In fact, DCs act as a link between the innate and the adaptive immune systems and can prime naïve T cells through the release of immune mediators and antigen presentation, thus determining the fate of the immune response [4]. 

Concerning the signaling pathways which are activated by the microbiota in order to modulate intestinal homeostasis, research was mainly focused on regulatory proteins and transcription factors. Now, the study of host microRNAs (miRNAs) as important regulators in the host–microbiota interplay is receiving great interest. miRNAs are small non-coding RNAs (20–25 nucleotides) that, after maturation, associate with proteins to form a microRNA silencing complex that post-transcriptionally regulates the expression of target messenger RNAs (mRNAs) by binding to complementary sequences in the 3’-UTR region [5]. This interaction either represses translation or triggers mRNA degradation [6]. Thus, miRNAs allow signaling pathways to be tightly regulated and are involved in the control of multiple cellular processes, including the immune response. Moreover, some studies reveal that both pathogens and gut bacteria greatly influence host miRNA expression [7,8,9].

The huge number of microbial cells represents a constant threat to the host. To maintain the spatial segregation of gut microbes and host tissues, the intestinal epithelium is covered by a mucus layer. The commensal microbiota resides in the outer mucus layer, while the inner layer is dense and impenetrable by bacteria, thus preventing the direct contact of the microbiota population with the underlying host cells [10,11]. Therefore, microbiota–host communication mainly involves secreted bacterial factors that can cross the inner mucus layer and interact with intestinal cells. In addition to secreted proteins, metabolites and quorum sensing autoinducers [12,13,14], bacteria also release extracellular membrane vesicles (MVs).

Bacterial extracellular MVs are nano-scale bilayer structures which are derived from the bacterial membranes. These membranous structures have a relevant role in interspecies communication, either between bacterial communities or between bacteria and their hosts. They enclose components of the cell membrane, periplasmic and cytosolic proteins, RNA, DNA and metabolites [15]. In fact, bacterial MVs serve as a secretion mechanism that allows the long-distance delivery of bacterial effectors in a protected environment. The study of gut microbiota-derived vesicles is an emerging topic. Today, there is considerable scientific evidence that extracellular MVs released by gut microbes are key players in microbiota–host communication. In the intestinal lumen, MVs released by the microbiota diffuse through the mucus layer and interact with epithelial and immune cells by means of their surface-associated MAMPS (extracellular polysaccharides and lipoproteins), which activate specific PRR-signaling cascades that modulate defense and immune responses. Moreover, the phagocytosis (by macrophages and DCs) and endocytic internalization (by epithelial cells) of microbiota MVs allow the intracellular delivery of bacterial peptidoglycan, RNA and DNA, which activate specific intracellular PRRs and the downstream regulatory pathways [16]. Components of bacterial MVs other than MAMPs can also modulate host responses. Thus, some effects mediated by microbiota MVs are strain-specific as they depend on the producer bacterial cell and the selected cargo. Proteomic and metabolomic approaches have revealed that MVs from probiotic and commensal strains enclose proteins and metabolites that aid bacterial survival in adverse gastrointestinal conditions and/or modulate the host immune system and intestinal epithelial barrier [17,18].

Previous studies conducted by our group have greatly contributed to proving that extracellular MVs mediate the effects of microbiota on the modulation of immune and defense responses in intestinal epithelial cells [19,20,21], in colonic explants [22] and in in vivo experimental models of colitis [23]. Our model of microbiota is *Escherichia coli*, specifically non-pathogenic intestinal strains such as the probiotic *E. coli* Nissle 1917 (EcN) and isolates from the stools of healthy human individuals. In this context, we recently showed that MVs are a mechanism used by the gut microbiota to steadily prime the innate immune system, activating DCs in a strain-specific manner [24]. The responses of the activated monocyte-derived dendritic cells (mo-DCs), in terms of the secreted cytokine profile and their ability to differentiate CD4^+^ T cells towards specific effector subsets, revealed that MVs from probiotic and commensal *E. coli* strains drive complex T helper (Th) responses that include a combination of Th2, Th22 and Th17. The main differences were observed in their ability to promote Th1 (pro-inflammatory, protective immunity towards intracellular pathogens) and T regulatory (T reg) (immune tolerance) responses. The Th1 response was mainly activated by MVs released by the probiotic EcN and other non-pathogenic B2 *E. coli* strains. In contrast, MVs secreted by the commensal ECOR12 (phylogenetic group A) did not trigger appropriate Th1 responses but promoted host tolerance against gut microbes by increasing the Treg/Th17 balance [24]. 

To further understand the differential activation of DCs by microbiota-derived MVs, here, we analyzed the miRNA profile induced by EcN and ECOR12 MVs in mo-DCs. Small RNA deep sequencing approaches allowed the identification of a common set of miRNAs which are modulated by MVs from both strains and miRNAs which are differentially expressed in response to EcN or ECOR12 MVs. An important group of identified miRNAs are related to immune function. Based on their differential expression and our subsequent validation experiments, we correlated some of the miRNAs with the cytokine profile and specific Th responses which were previously reported for both microbiota strains.

## 2. Materials and Methods

### 2.1. Bacterial Strains and Growth Conditions

The probiotic EcN (serotype O6:K5:H1) was provided by Ardeypharm (GmbH, Herdecke, Germany). ECOR12 is a commensal strain isolated from the fecal samples of a healthy human adult and is included in the *Escherichia coli* ECOR reference collection [25]. Strains were grown aerobically at 37°C in Luria–Bertani broth (LB). Growth was monitored by measuring the optical density at 600 nm.

### 2.2. Isolation of Membrane Vesicles

MVs were isolated as previously described [17]. Briefly, overnight cultures were centrifuged at 10,000*× g*, for 20 min at 4 °C, and bacterial pellets were discarded. The culture supernatants were then filtered through a 0.22 μm pore filter (Merck Millipore) and concentrated using Centricon Plus-70 centrifugal filter units, with a 10 kDa cutoff (Merck Millipore). The concentrated supernatants were further filtered to remove any remaining bacteria. MVs were collected by centrifugation at 150,000*× g* for 1 h at 4 °C and washed and resuspended in phosphate buffer saline (PBS). Sterility was confirmed on LB plates and the quality of the MVs was assessed by cryo-transmission electron microscopy, as described previously [24]. The MVs were quantified by measuring protein concentration, and the aliquots were stored at −20 °C until use.

### 2.3. Generation and Stimulation of Human Monocyte-Derived DCs

Human mo-DCs were generated in vitro, as described previously [24]. Peripheral blood mononuclear cells (PBMCs) were isolated from the fresh buffy coats of healthy donors by density gradient centrifugation using Histopaque 1077 (Sigma-Aldrich Chemical Co, San Luis, MO, USA). Monocytes were then captured with magnetic CD14 microbeads (Miltenyi Biotec, Bergisch Gladbach, Germany), following the manufacturer’s protocol. To generate immature DCs (iDCs), the monocytes were seeded at a density of 2 × 10^6^ cells/mL in 6-well plates and cultured for 6 days (37 °C, 5% CO_2_) in an mo-DC differentiation medium that contained 800 IU/mL granulocyte-macrophage colony–stimulating factor. (GM-CSF) and 1000 IU/mL IL-4 (Miltenyi Biotec). The fresh complete medium was added on the fourth day of culture.

Before stimulation with bacterial MVs, the generated iDCs were washed and kept in fresh mo-DC differentiation medium for at least one hour at 37 °C in 5% CO_2_. Next, MVs were added at a concentration of 10 μg/mL and cells were incubated for 24 h. The stimulation conditions (incubation time and MV amount) were kept identical to those used in the previous study [24]. Unstimulated cells received the same volume of PBS and were incubated in parallel as a control. Mature DCs (mDCs) were collected and processed for RNA isolation. The culture supernatants were sterile filtered through a 0.2 µm pore filter and stored at −80 °C for cytokine quantification.

### 2.4. Flow Cytometry Analysis of Surface Markers

Maturation of the vesicle-stimulated mo-DCs was verified by flow cytometry using the MO-DC Differentiation Inspector Kit (Miltenyi Biotec), as described previously [24]. At least 10,000 events were acquired on a Gallios flow cytometer (Beckman Coulter, Inc., Fullerton, CA, USA) with a 3-laser/10 color standard configuration. The specific fluorescence intensity was quantified as the mean fluorescence intensity (MFI), calculated by subtracting the background of isotype-matched control staining from the total fluorescence.

### 2.5. RNA Isolation

RNA was isolated from the mo-DCs using the Qiagen RNeasy Mini Kit, according to the manufacturer’s instructions (Qiagen). RNA concentration was measured using a NanoDrop TM-2000 spectrophotometer (Thermo Fisher Scientific, Waltham, MA, USA). The quality of the RNA was evaluated on an Agilent 2100 Bioanalyzer (Agilent Technologies, Inc., Santa Clara, CA, USA) to obtain the RNA integrity number (RIN). All samples had RIN values above 9.1.

### 2.6. Small RNA-seq Library Preparation and Sequencing

The Illumina TruSeq small RNA Sample Prep Kit (ref. RS-200-0012) was used according to the manufacturer’s protocol. About 500 ng of total RNA was processed for each of the nine samples. First, 3′-adapters, and, subsequently, 5′-adapters, were ligated to the RNA. cDNA was synthesized using reverse transcriptase (SuperScript II, ref. 18064-014, Invitrogen, Thermo-Fisher, Waltham, MA, USA) and a specific primer (RNA RT Primer) complementary to the 3′-RNA adapters. cDNA was further amplified by PCR, using the indexed adapters supplied in the kit. Finally, libraries were size selected using 6% Novex^®^ TBE Gels (ref. EC6265BOX, Life Technologies, Carlsbad, CA, USA. Fragments with insert sizes of 18 to 36 bp were cut from the gel, and the DNA was precipitated and eluted in 10 µl elution buffer.

The final libraries were analyzed using an Agilent DNA 1000 chip to estimate the quantity and check the size distribution and were then quantified by qPCR using the KAPA Library Quantification Kit (ref. KK4835, Kapa Biosystems, Basilea, Switzerland) prior to amplification with Illumina’s cBot. The libraries were pooled and loaded at a concentration of 10 pM onto the flowcell and were sequenced using a 50-cycle single-end protocol on an Illumina HiSeq 2500.

### 2.7. Small RNA-seq Pre-Processing and Differential Expression Analysis

Quality control was assessed with FastQC (v0.11.7) [26]. Adaptor trimming from the 3′-end was performed with Skewer (v0.2.2) [27]. Trimmed reads were then aligned with bowtie2 (v 1.2.2) [28], with the “-non-deterministic” parameter, on an hg38 version of the Homo sapiens genome.

Quality control of the mapped reads (BAM files) was performed with Qualimap (v2.2.1) [29]. Reads were assigned to miRNAs with htseq-count (v0.9.1) [30], using version 22 of the miRBase annotation [31]. The raw and processed data are available in The Gene Expression Omnibus (GEO) database under accession number: GSE144355.

The R/Bioconductor [32,33] DESeq2 (v1.20.0) package [34] was used to calculate the pairwise differential expression of miRNAs between the experimental groups. The DESeq2 method assesses differential expression using the Wald statistical test. The principal component analysis was performed with the prcomp function of the stats R package. Graphics related to the small RNA-sequencing data analysis were plotted using R packages: ggplot2 (v3.0.1) [35,36], pheatmap (v1.0.12) [37] and VennDiagram (v1.6.20) [38].

### 2.8. Gene Ontology Enrichment Analysis of miRNA Targets

Three miRNA target databases were browsed and filtered for the analysis: miRDB (v6.0) [39,40], TargetScan (v7.2) [41] and miRTarBase (v7.0) [42]. The miRDB targets were filtered using a target prediction score of >80, following the authors’ recommendations. The TargetScan targets were filtered using a “context++” score of <−0.25, which represents the predicted efficacy of repression. Finally, only the miRTarBase targets that showed a functional MTI (miRNA–Target Interactions) with strong support were kept.

We next retrieved the miRNAs upregulated in both the EcN MV and ECOR12 MV groups (compared to the control), based on the selection of false discovery rate (FDR)-adjusted *p*-values (<0.005) and of log2 fold changes > 1.5 in both comparisons. Targets for these top miRNAs were retrieved from the three databases. A target for a specific miRNA was kept if it was found in at least two out of the three databases. The lists of targets of each miRNA were analyzed for gene ontology (GO) [43,44] enrichment using the “GOstats” [45] R/Bioconductor package, with a hypergeometric test *p*-value cutoff of 0.01. Prior to running the GO enrichment analysis, the target gene symbols were converted to “Entrez gene IDs” with the biomaRt R package [46]. The GO enrichment analysis was performed on each of the miRNA target lists independently, against the three GO aspects: biological process (BP), molecular function (MF) and cellular component (CC). We kept the ontologies that were found to be enriched in at least three of the miRNA target lists. Ontologies were further grouped into more general categories, using the QuickGO tool as a guide [47].

### 2.9. Validation of miRNAs and Predicted mRNA Targets by Quantitative Reverse Transcription-PCR (RT-qPCR)

For miRNA validation, RNA (5 ng/μL) was reverse transcribed using a miRCURY LNA™ RT kit (Qiagen) in a final volume of 10 µL. The quantification of specific miRNAs was performed by real-time PCR, using miRCURY LNA™ miRNA PCR Assays (Qiagen) with gene-specific primers (Supplementary Table S1), according to the manufacturer’s protocol. The miRNAs has-miR-421, has-miR-let-7f-5p (both identified in the RNA-seq analysis as unregulated miRNAs) and the stable RNU6-1 reference gene were used for normalization.

For the expression analysis of the target mRNAs, cDNA synthesis was performed using the High Capacity cDNA Reverse Transcription kit (Applied Biosystems, Foster City, CA, USA) in a final volume of 20 µL. Quantitative PCR was performed on a StepOne Plus PCR cycler (Applied Biosystems), with the SYBR^®^ Green PCR Master Mix (Applied Biosystems) and specific oligonucleotides for the selected genes (Table 1). The housekeeping HPRT-1 gene was used as a normalizing gene. Expression of IL-12 was analyzed with specific Taqman probe and primers (Applied Biosystems). The standard PCR program consisted of one denaturation cycle for 10 min at 95 °C, followed by 40 cycles of 15 s at 95 °C and 1 min at 60 °C. In all assays, qPCR was performed on a StepOne Plus PCR cycler (Applied Biosystems Relative gene expression was calculated as the fold change compared with the control and calculated by means of the 2^−ΔΔCt^ formula [48].

### 2.10. Cytokine Quantification

Secreted IFN-γ, IL-12 and IL-10 were quantified in culture supernatants by the human ProcartaPlex Immunoassay (eBiosciences Inc., Santa Clara, CA, USA), according to the manufacturer’s instructions. The concentration of each analyte was detected using the MAGPIX instrument (Luminex Corp., Austin, TX, USA), using the facilities of the Scientific and Technological Centers of the University of Barcelona (CCiT-UB, Barcelona, Spain). The results were analyzed with xPONENT^®^ 4.2 software (Luminex Corp.).

### 2.11. Statistical Analysis

Statistical analysis was performed using GraphPad Prism 7.0 software (GraphPad, San Diego, CA, USA). The cytokine and RT-qPCR data were taken from at least three independent biological experiments performed in triplicate and were expressed as the mean ± standard error of the mean (SEM). The differences between more than two groups were assessed using one-way ANOVA, followed by Bonferroni’s test. Significant differences were established at *p*-value ≤ 0.05.

### 2.12. Ethics

Buffy coats from healthy donors were provided by the “Banc de Sang i Teixits” of Barcelona, according to the signed agreement with the Institution. The use of anonymous, non-identifiable human samples was approved by the Bioethics Committee of the University of Barcelona (Institutional Review Board: 1R800003099).

## 3. Results

### 3.1. miRNA Expression Profiling

The previous results of our group showed the differential activation of mo-DCs by MVs from the microbiota *E. coli* strains EcN and ECOR12 [24]. To gain new insights into the regulatory mechanisms activated by microbiota-derived vesicles, miRNA expression was evaluated by high-throughput sequencing (deep sequencing) analysis using the Illumina platform. Human mo-DCs were stimulated for 24 h with MVs (10 μg/mL) isolated from the probiotic EcN and the commensal ECOR12. Untreated mo-DCs were used as a control. Data were obtained from three independent experiments, each performed with DCs derived from at least two donors. The maturation of the mo-DCs was assessed by flow cytometry analysis using the MO-DC differentiation inspector kit. As expected, both ECOR12 and EcN MVs induced high levels of the maturation surface marker CD83 and reduced the expression of CD209 (DC-SING) compared to the untreated mo-DCs (Supplementary Figure S1).

**Figure 1:**
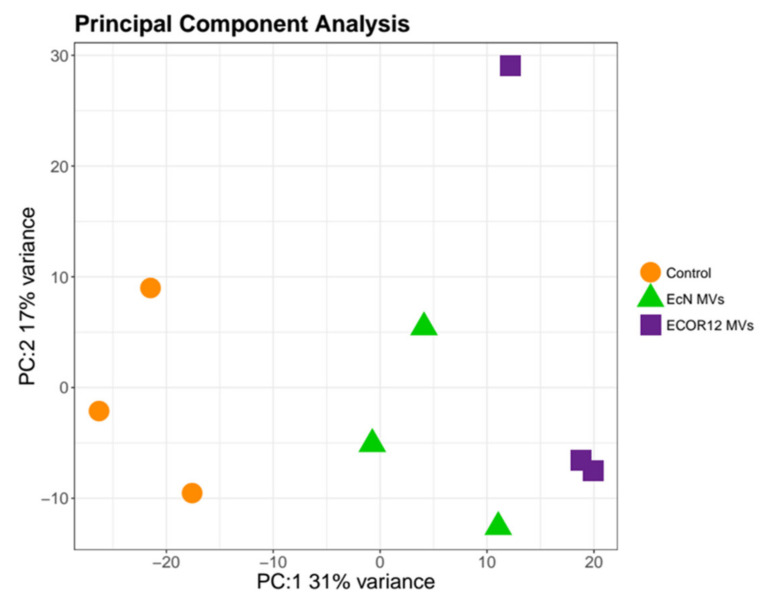
Principal component analysis of the small RNA-seq samples, calculated using DESeq2-normalized counts per microRNA (miRNA).

Samples from the three experimental groups (EcN MVs, ECOR12 MVs and control) were sequenced in triplicate. A summary of the number of reads and mapping statistics is available in Table 2. Briefly, all samples reached a unique mapping rate between 68.2% and 84.5%. A principal component analysis based on the normalized read counts showed that the samples were grouped as expected (Figure 1). The raw and processed data are available in GEO under accession number: GSE144355.

### 3.2. Differential Expression of miRNAs in Response to Probiotic and Commensal-Derived MVs

In a comparison between the samples, miRNAs were selected as differentially expressed when an FDR-adjusted *p*-value under 0.005 was obtained, along with an absolute log2 fold change greater than 0.6 (Supplementary Table S2). The FDRadjustment is the standard multiple testing correction applied by the widely used “DESeq2” method. Selecting miRNAs using a stringent FDR-adjusted *p*-value threshold allowed us to reduce the chance of finding false positives in the selected results. Setting the FDR cutoff as <0.005 meant that the proportion of false positives that we expected among differentially expressed miRNAs was 0.5%. The additional log2 fold change threshold was added to narrow down the list of miRNAs of interest to those that had an increase in expression of at least −50%. A log2 fold change of 0.6 corresponds to a linear fold change of 1.5, and this means a 50% increase in expression.

**Figure 2:**
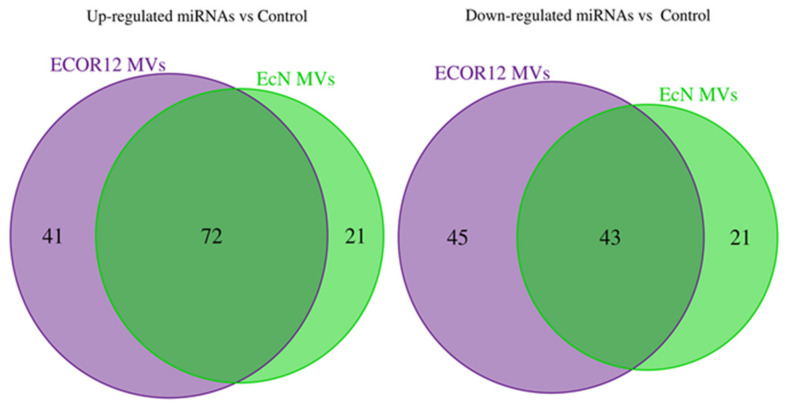
Venn diagrams representing the comparative number of differentially expressed miRNAs in dendritic cells (DCs) treated with *E. coli* Nissle 1917 (EcN) membrane vesicles (MVs) or ECOR12 MVs. miRNAs were selected based on FDR-adjusted *p*-value < 0.005 and log2 fold change >0.6 for the upregulated miRNAs or <−0.6 for the downregulated miRNAs.

**Figure 3:**
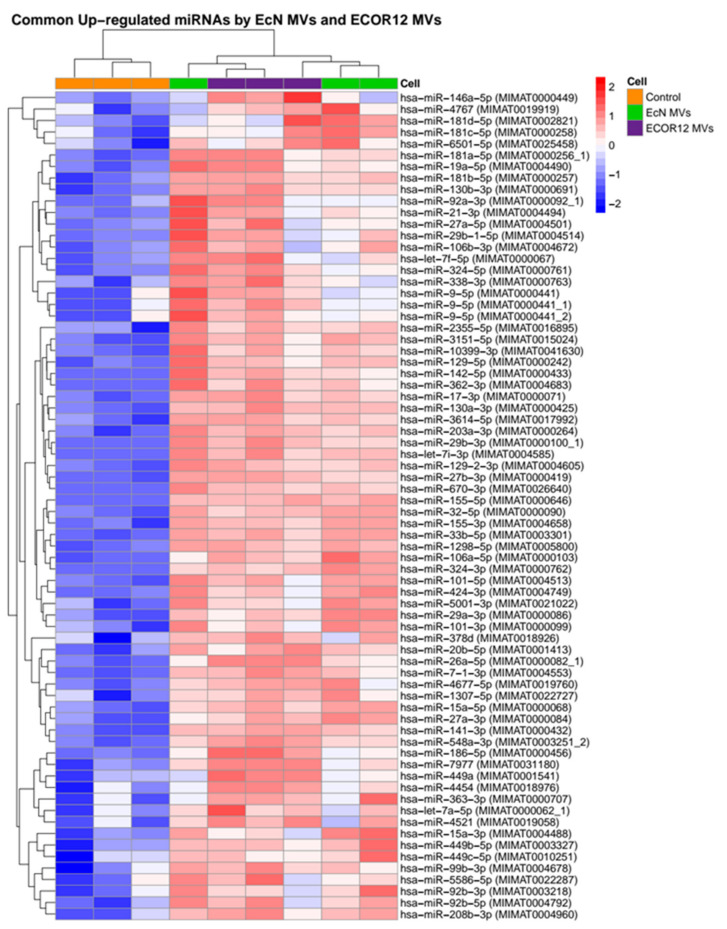
**Heatmaps of common miRNAs upregulated by both EcN MVs and ECOR12 MVs compared to the control untreated DC group.** The color scale next to the panel illustrates the relative expression levels of the indicated miRNAs in each sample. Upregulated miRNAs were selected based on FDR-adjusted *p*-value <0.005 and log2 fold change > 0.6.

**Figure 4:**
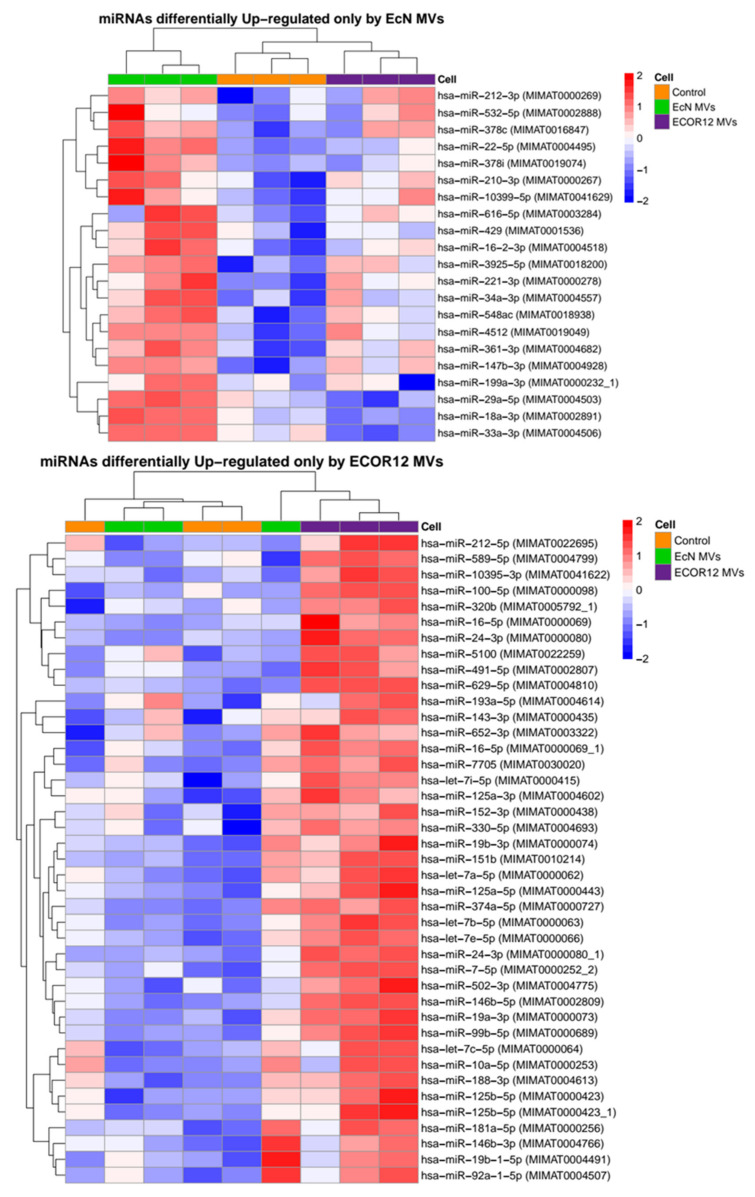
Heatmaps of miRNAs differentially upregulated by EcN MVs or ECOR12 MVs compared to the control untreated DC group. The color scale next to each panel illustrates the relative expression levels of the indicated miRNAs in each sample. Upregulated miRNAs were selected based on FDR-adjusted *p*-value < 0.005 and log2 fold change > 0.6.

Using this selection criteria, 157 miRNAs were found to be deregulated (93 upregulated and 64 downregulated) in DCs that were challenged with MVs from the probiotic EcN when compared to the control samples, and 201 were found to be deregulated (113 upregulated and 88 downregulated) in cells treated with MVs from the commensal ECOR12 when compared to the control samples. The analysis revealed a common set of miRNAs modulated by MVs from both strains (43 downregulated, 72 upregulated) (Figure 2). Heatmaps showed the expression profiles for miRNAs upregulated by EcN and ECOR12 MVs in each of the three independent samples (Figure 3 and Figure 4). The equivalent information concerning the differentially downregulated miRNAs is presented as Supplementary Material (Figures S2 and S3).

### 3.3. Selection of miRNAs

**Figure 5:**
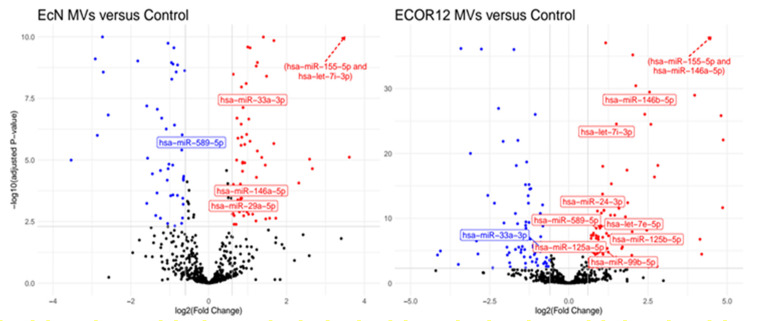
Volcano plots of miRNA expression in DCs challenged with EcN MVs (left) or ECOR12 MVs (right) compared to the control group, as calculated by DESeq2. Color highlighted dots correspond to downregulated (blue) or upregulated (red) miRNAs based on an FDR-adjusted *p*-value < 0.005 and an absolute log2 fold change > 0.6. Labeled miRNAs are those selected for RT-qPCR validation. To make the plot area more readable, the upper x axis scale was cut off from the plot. Selected upregulated miRNAs that fit into the deleted area are indicated in parentheses.

Twelve miRNAs were selected for RT-qPCR validation (Figure 5). Ten were closely related to DC maturation and immune responses (see the Discussion) and covered common upregulated miRNAs (miR-155-5p, let-7i-3p and miR-146a-5p) and miRNAs that were differentially upregulated by ECOR12 or EcN MVs (miR-125b-5p, cluster miR-125a-miR-99b-5p-let7e-5p, miR-24-3p, miR-146b-5p and miRNA-29a-5p). Some of the differentially expressed miRNAs may explain the cytokine profile and derived specific Th responses which were previously described in mo-DCs activated by these microbiota-derived vesicles [24]. The other two miRNAs displayed opposite regulation in response to EcN MVs or ECOR12 MVs (miR-33a-3p, miR-589-5p). Detailed information on the expression and significance of these selected miRNAs is provided in Table 3.

### 3.4. Validation of Selected miRNAs

**Figure 6:**
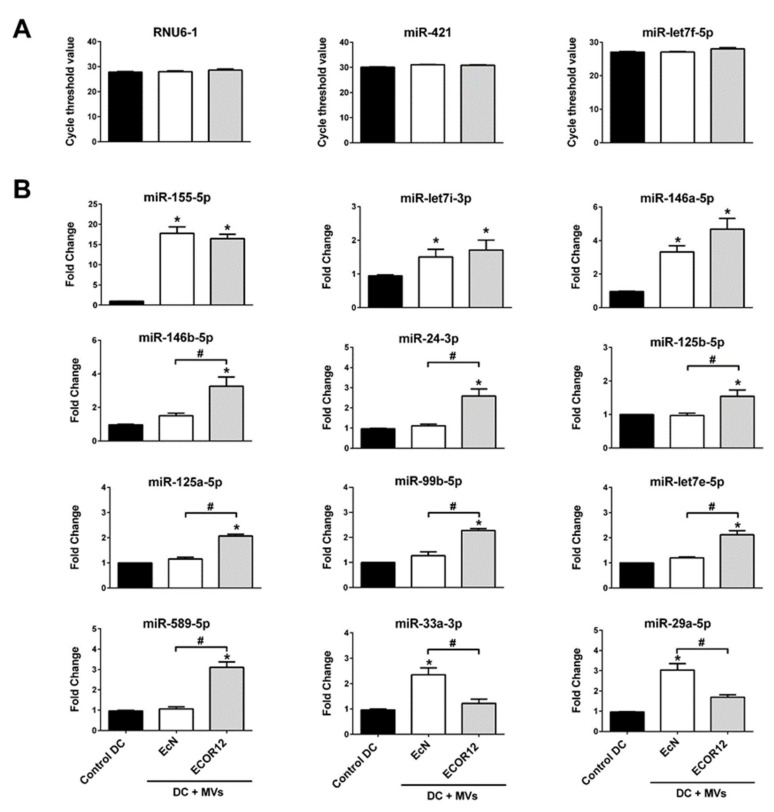
**RT-qPCR validation of selected miRNAs**. Immature monocyte-derived dendritic cells (mo-DCs) were challenged for 24 h with MVs (10 µg/mL) from EcN (white bars) or ECOR12 (gray bars). Untreated control DCs are shown as black bars. (**A**) Cycle-threshold values for the reference genes (RNU-6, miR-421 and miR-let7f-5p). (**B**) Relative miRNA levels were measured by RT-qPCR and normalized to the three indicated reference genes. Data (mean ± SEM) are from six independent biological experiments (six donors) performed in triplicate and are presented as fold changes compared to untreated control cells. Statistical differences were evaluated by one-way ANOVA, followed by Bonferroni’s test (*p* < 0.05). * Significance against untreated control cells; ^#^ significance between cells stimulated with EcN MVs or ECOR12 MVs.

Monocytes isolated from six independent donors were differentiated into mo-DCs and stimulated with EcN or ECOR12 for 24 h. RNA was isolated and standard miRCURY LNA™ miRNA RT-PCR assays were performed using gene-specific primers (Qiagen), as described in the Methods. Expression was normalized according to three stable miRNAs, the housekeeping RNU6-1 gene and two stable miRNA genes whose expression remained unchanged between the control and vesicle-stimulated DCs in the three independent RNA-seq analyses (Figure 6A).

The results showed a good correlation with the RNA-seq data, except for miR-33a-3p and miR-589-5p. For these mRNAs, the RT-qPCR results confirmed the differential specific upregulation of both miRNAs (*p* = 0.0002, *p* = 0.0001) but failed to prove the downregulation of miR33a-3p by the ECOR12 MVs and the downregulation of miR589-5p by the EcN MVs (Figure 6B). Under these conditions, the expression levels of both miRNAs were close to those of the untreated control DCs. Concerning the commonly upregulated miRNAs, the RT-qPCR results corroborated the notion that EcN and ECOR12 MVs elicit the upregulation of miR-155-5p and let-7i with comparable fold change expression values between strains. The data also confirmed that miR-146a-5p was differentially upregulated by these microbiota-derived MVs with statistical significance (about three-fold by EcN MVs and six-fold by ECOR12 MVs). The RT-qPCR analysis also validated the specific upregulation of miR-125b-5p, miR-125a-miR-99b-5p-let7e-5p, miR-24-3p and miR146b-5p by ECOR12 MVs and miR29-5p by EcN MVs, with fold changes that were well-matched with those obtained from the RNA-seq data (Figure 6B).

### 3.5. Expression Analysis of Selected miRNA Target Genes Relevant for DC Maturation and Function

As miRNAs are relevant players in post-transcriptional regulation, we analyzed the mRNA levels of genes which are known to be targeted by the selected miRNAs (Table 4). The analysis was mainly focused on maturation and immune-related genes that were already experimentally validated as targets of the selected miRNAs.

**Figure 7:**
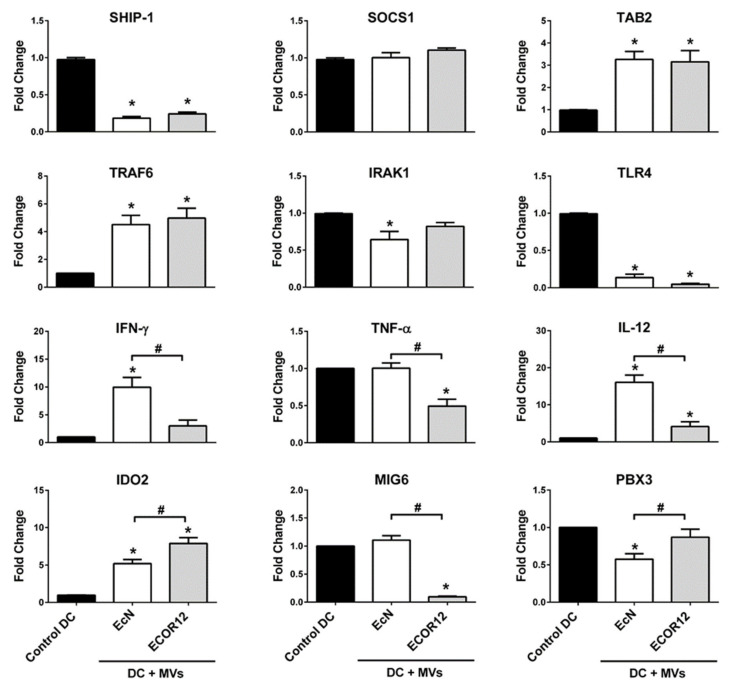
**RT-qPCR expression analysis of miRNA target genes**. Immature mo-DCs were challenged for 24 h with MVs (10 μg/mL) from EcN (white bars) or ECOR12 (gray bars). Untreated control DCs are shown as black bars. Relative mRNA levels of the indicated genes were measured by RT-qPCR, using HPRT-1 as the reference gene. Data (mean ± SEM) are presented as fold changes compared to untreated control cells (six independent biological experiments, performed in triplicate). Statistical differences were evaluated by one-way ANOVA, followed by Bonferroni’s test (*p* < 0.05). * Significance against untreated control cells; ^#^ significance between cells stimulated with EcN MVs or ECOR12 MVs.

The relative mRNA levels of the target genes which are indicated in Table 4 were assessed by RT-qPCR in mo-DCs that were treated with EcN or ECOR12 MVs for 24 h. The results showed a good negative correlation between the miRNA and the corresponding gene target(s), except for miR-155-5p and its targets, SOCS1 and TAB2, and for miR-146 and its target, TRAF6 (Figure 7). Notably, the other known target genes of common vesicle-upregulated miRNAs matched the expected expression pattern. In this sense, the downregulation of SHIP1 and TLR4 in DCs that were challenged by either EcN MVs or ECOR12 MVs correlated with the increased miR-155-5p and miR-146-a-5p expression (Figure 7).

**Figure 8:**
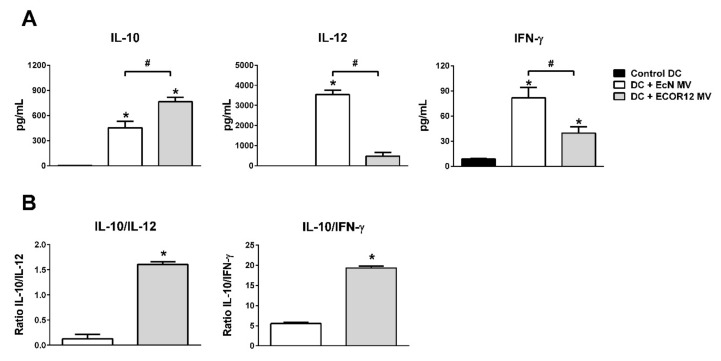
Quantification of secreted INF-γ, IL-12 and IL-10 by MV-stimulated DCs. (**A**) Cytokines were quantified in DC-culture supernatants after 24 h stimulation with EcN MVs (white bars) or ECOR12 MVs (grey bars). Untreated mo-DCs were kept in DC medium as a control (black bars). Data (mean ± SEM) are from four independent biological experiments which were performed in triplicate. (**B**) Ratio IL-10/IL-12 and IL-10/INF-γ values. Statistical differences were evaluated by one-way ANOVA, followed by Bonferroni’s test (*p* < 0.05). * Significance versus control DCs, # significance between cells stimulated with EcN MVs or ECOR12 MVs.

The differential upregulation of miRNAs miR-24-3p, miR125b-5p and the miR-125a-let-7e-5p cluster by ECOR12 MVs correlated with the reduced mRNA levels of the pro-inflammatory cytokines IFN-γ, TNF-α and IL-12 (Figure 7). Consistently, the levels of secreted IFN-**γ** and IL-12 cytokines were significantly lower in the DCs that were exposed to ECOR12 MVs than in the EcN-treated DCs (Figure 8A). The secretion of these pro-inflammatory cytokines inversely correlated with IL-10 secretion. The IL-10/IL-12 and IL-10/INF-γ ratios were higher for ECOR12 vesicle-treated DCs (Figure 8B). The ability of DCs to induce tolerogenic responses is essential for the maintenance of immune homeostasis. The expression of indoleamine 2,3-dioxygenase enzymes (IDO) is among the mechanisms which are used by DCs to generate T regulatory responses. Human DCs express two IDO enzymes, IDO1 and IDO2, which display different expression and regulatory patterns [58]. In this context, IDO2 has been identified as a potential target of miRNA-29a-5p (upregulated by EcN MVs). RT-qPCR analysis revealed that although IDO2 was upregulated in DCs that were incubated with either ECOR12 or EcN vesicles, the level of expression was significantly lower in EcN-stimulated cells (Figure 7).

To further corroborate the differential expression of miR-33a-3p and miR-589-5p as elicited by these microbiota-derived MVs, the mRNA levels of known target genes were measured. The reduced expression of PBX3 in the mo-DCs stimulated by EcN MVs and that of MIG6 in cells stimulated by ECOR12 MVs correlates with the differential upregulation profile of the corresponding miRNAs in response to these MVs (Figure 7).

**Figure 9:**
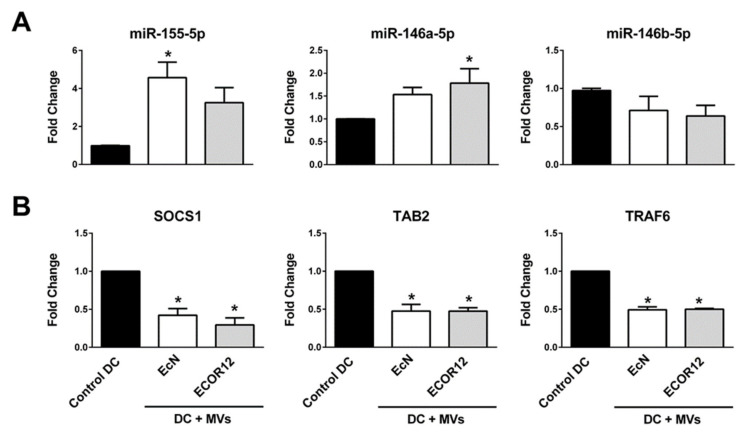
Correlation between expression levels of miR-155-5p, miR-146a-5p and miR-146b-5p and their targets at a short time post-stimulation. Immature mo-DCs were stimulated with EcN or ECOR12 MVs for 6 h. (**A**) Relative levels of the indicated miRNAs and (**B**) relative levels of the known target mRNAs, SOCS1, TAB2 (miR-155-5p) and TRAF (miR-146-5p), were assessed by RT-qPCR and normalized, as described in Figure 6 and Figure 7’s legends, respectively. Data are presented as fold changes compared to untreated control cells (four independent biological experiments which were performed in triplicate). Statistical differences were evaluated by one-way ANOVA, followed by Bonferroni’s test. * Significance against untreated control cells.

Concerning the unmatched target/miRNA pairs SOCS1-TAB2/miR-155-5p and TRAF6/miR-146-5p, we sought to analyze their expression at a short time post-stimulation. These miRNAs are relevant for DC maturation and their activation by lipopolysaccharide (LPS), or pathogenic bacteria starts to be apparent at 6 h post-infection [59]. To perform this analysis, mo-DCs (four independent healthy donors) were stimulated with EcN or ECOR12 MVs for 6 h. The expression of all of the selected miRNAs was assessed by RT-qPCR. At this incubation time, only miR-146a-5p and miR155-5p showed increased expression compared to the unstimulated mo-DCs (Figure 9). Particularly, at 6 h of incubation with MVs, miR-146a-5p expression was about half of that achieved after 24 h of stimulation, whereas miR-146b-5p expression remained as in the control cells. For miR-155-5p, the activation elicited by MVs at 6 h (approximately a four-fold increase) was 25% of the activation reached at 24 h (18-fold). The other miRNAs were not activated at this early time, displaying expression levels comparable to those of the control cells. At this early time of incubation, the mRNA levels of the direct target genes SOCS1, TAB2 and TRAF6 were significantly reduced. The fact that the levels of these target mRNAs were counteracted or even increased at 24 h of incubation suggests a hierarchy in miRNA expression and action pointing to other compensatory mechanisms being activated during the DC maturation/activation processes.

### 3.6. miRNA Target Functional Analysis

In addition to immune related genes, we sought to explore other genes that might be modulated in DCs by the miRNAs identified in the present study in response to microbiota-secreted extracellular vesicles. To this end, functional gene ontology (GO) enrichment analysis was performed for up-regulated miRNAs in both the EcN MV and ECOR12 MV groups compared to the control. After the selection and filtering of the three databases, we worked on the following total number of targets for human miRNAs: 812,240 for miRDB, 63,113 for TargetScan and 10,753 for miRTarBase.

**Figure 10:**
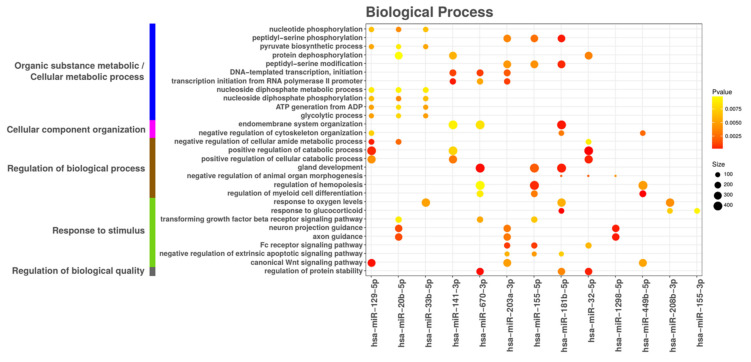
**Enriched biological processes (gene ontologies) of the 23 most commonly upregulated miRNA targets**. Analysis was based on the 23 miRNAs upregulated by both EcN and ECOR12 MVs when compared to the control untreated DC group. The size of the dots corresponds to the number of genes in the ontology and the color to the GOstats hypergeometric test *p*-value. The ontologies are shown only if they were found to be enriched in at least three of the miRNA target lists.

The number of targets found for each of the 23 selected miRNAs (based on FDR-adjusted *p*-value < 0.005 and log2 fold changes > 1.5 in both comparisons) and per database can be found in Supplementary Table S3. A total of 897, 104 and 58 ontologies were found to be enriched for the BP, MF and CC classes, respectively. After the selection of the ontologies that were enriched in at least three miRNA target lists, this number was further reduced to 29 for BP, 1 for MF and 2 for CC (Figure 10 and Supplementary Figure S4).

The 29 BP ontologies were mapped onto the GO tree (Supplementary Figure S5) and grouped into more general processes. The predicted targets of the most commonly vesicle-upregulated genes are mainly involved in the activity and regulation of metabolic and energy-producing pathways, the organization of the cytoskeleton and endomembranous structures (relevant in the context of phagocytic cells) and the cell response to external stimulus (Figure 10). The MF and CC ontologies mainly included functions related to transcriptional regulation (Supplementary Figure S4).

## 4. Discussion

Accumulating evidence from the last few years shows that the intestinal microbiota plays a major role in health and disease in humans. Changes in the microbiota composition and diversity have been described in various nutritional imbalances and diseases [60,61,62]. Microbiota–host communication is mainly achieved by secreted mediators that can diffuse across the inner mucus layer. In this context, MVs released by the gut microbiota are part of the bacteria–host molecular dialog, allowing the delivery of factors that prime the host’s innate immune system.

We have recently shown that extracellular vesicles from probiotic and gut resident *Escherichia coli* strains differentially activate mo-DCs towards Treg or Th responses [24]. Importantly, MVs from these strains enclose identical MAMPs, such as LPS (TLR4 ligand), lipoproteins (TLR2 ligand), peptidoglycan (NOD1/NOD2 ligand) DNA and RNA (TLR9 and TLR7 ligands), which trigger the same signaling pathways that lead to the upregulation of antimicrobial peptides and inflammatory cytokines. However, they activate DCs in a strain-specific manner, thus indicating that other cargo molecules account for the specific regulatory effects. We showed that extracellular vesicles from commensal and probiotic *E. coli* strains elicit similar Th17 and Th22 responses, but they significantly differ in the extent of their Th1- and Treg-driven responses. MVs from probiotic EcN (group B) activate mo-DCs towards a robust Th1 response, whereas vesicles from commensal ECOR12 (group A) induce an overall outcome that is dominated by a tolerogenic immune response [24].

In order to gain new insights into the regulatory mechanisms used by gut beneficial microbes to specifically prime and modulate DCs and derive host immune responses, here, we have used an RNA deep sequencing approach to identify differentially expressed miRNAs in DCs which have been challenged with the MVs of EcN or ECOR12. The results showed that, following 24 h of incubation, a set of miRNAs was modulated in the same direction by MVs from both EcN and ECOR12 strains (43 downregulated, 72 upregulated). Functional GO enrichment analysis of the putative target genes of the 23 miRNAs commonly upregulated in the two treated groups revealed that MVs from both gut resident strains modulate basic biological processes, including the metabolism of glucose, amino acids and nucleotides; the organization of cellular and membrane components which are essential in phagocytic and antigen-presenting cells; and signaling pathways that control cell growth and development.

This commonly upregulated group also includes miRNAs that are known to be expressed following DC activation and maturation through TLR stimuli such as miR-155, miR-let7i and miR-146a [50]. The microRNAs miR-155 and miR-146a have been widely studied as key regulators of the immune system [63]. The expression of these miRNAs in DCs is upregulated by inflammatory stimuli such as pro-inflammatory cytokines (TNF-α, IFN-γ) and/or TLR ligands (LPS, dsDNA). Their function is to modulate the inflammatory response by targeting the effectors of the TLR pathways. MicroRNA-155 is required for proper DC function. Mice that were deficient in miR-155 showed an impaired ability to trigger appropriate protective immune responses against pathogens, and DCs lacking miR-155 are unable to prime a functional T cell response [64]. In fact, miR-155 is considered a multifunctional regulator of innate immunity. The activation of DCs by TLR ligands leads to a cascade of pro-inflammatory cytokines. By targeting the gene transcripts of these signaling pathways (IKK in the NF-kB pathway and TAB2 in the TLR/IL-1 pathways), miR-155 exerts a negative feed-back loop, limiting the exacerbated secretion of pro-inflammatory cytokines. However, miR-155 also functions as a pro-inflammatory mediator by targeting the SOCS1 transcript [50], a protein that negatively regulates cytokine receptors’ downstream pathways in order to attenuate cytokine signaling. SOCS1 is also a negative regulator of the antigen-presenting capacity of DCs [65]. Other functions of miR-155 in DCs are linked to the expression of co-receptor molecules that mediate pathogen binding. In this context, the upregulation of miR-155 reduces the expression of the C-type lectin receptor DC-SING by directly targeting the transcription factor PU.1. The phosphoinositide phosphatase SHIP1 is also directly regulated at the post-transcriptional level by miR-155 [49]. SHIP1 is a multifunctional regulator of immune cell activation that influences signaling pathways through mechanisms that depend on its phosphatase activity and mechanisms that are activity-independent [66]. The pleiotropic, even opposite, effects of miR-155 indicate its relevant role in the fine-tuning of DC function. Consistent with the transcriptional activation of miR-155 by LPS and other TLR ligands, our RNA-seq and RT-qPCR results confirmed the upregulation of this miRNA by MVs from both gut resident *E. coli* strains. Increased levels of miR-155-5p were already detected at 6 h of stimulation and reached high expression at 24 h. As expected, high miR-155-5p levels correlated with a reduced expression of DC-SIGN (maturation marker CD209 in Supplementary Figure S1). Expression analysis of the direct miR-155-5p targets revealed a complex hierarchy that is compatible with the pleiotropic and multifunctional role of miR-155-5p [67]. The downregulation of SOCS1 and TAB2 transcripts was only apparent early on in the vesicle stimulation, whereas SHIP1 mRNA levels were significantly reduced at 24 h. Several factors influence the complex regulatory networks and compensatory mechanisms operating during DC maturation/ activation processes: (i) certain miRNAs can regulate the expression of other miRNAs, (ii) a single miRNA has multiple targets and (iii) a single transcript target can be regulated by different miRNAs. For instance, SOCS1 is also a direct target of miR-let7i-3p, a miRNA which is upregulated during DC activation by *E. coli* MVs. This miRNA blocks translation without altering SOCS1 mRNA levels [51].

miR-146a-5p is among the commonly upregulated miRNAs which were identified by RNA-seq in this study, although its expression differs depending on the vesicle producer strain. Both RNA-seq and RT-qPCR data showed that miR-146a-5p expression levels were significantly higher in DCs that were challenged with ECOR12 MVs than in cells exposed to EcN MVs. The expression of miR-146a-5p is transcriptionally induced by NF-κB in response to TLR activation (LPS) and acts a negative regulator of inflammation by silencing TRAF6 and IRAK, key adaptor proteins of the NF-κB signaling pathway. Thus, miRNA-146a acts as a negative feedback loop that leads to the inhibition of NF-κB activation in order to guarantee a dynamic balance between anti- and pro-inflammatory signals [52]. In addition, miR146 has been proposed to mediate endotoxin tolerance, a state of low responsiveness to subsequent LPS challenges. This mechanism is related to the miR-146-mediated blockage of signaling that attenuates the secretion of pro-inflammatory cytokines. It has been proposed that reduced levels of IRAK1 protein might account for the refractory behavior of immune cells against new LPS doses [68]. The human genome encodes two copies of miR-146, miR-146a and 146b, which only differ by two nucleotides at the 3′-end. Due to this high sequence similarity, both miR-146 copies target the same set of gene transcripts. Importantly, genes encoding miR-146a and miR146b are in different chromosomes and their expression responds to different stimuli. Whereas the LPS-mediated induction of miR-146a depends on NF-κB, the upregulation of miR-146b depends on IL-10 and occurs via the transcription factor STAT3 [69]. The induction of miR-146b by LPS displays delayed kinetics with respect to miR146a, since it depends on the IL-10 production that follows an LPS challenge. IL-10 is an anti-inflammatory cytokine that plays an essential role in inhibiting immune responses and in preventing prolonged inflammatory conditions. Interestingly, our results show that miR146b-5p is upregulated by ECOR12 MVs but not by EcN MVs. Moreover, miR-146b induction requires longer exposure times than miR-146a induction. The differential expression of miR-146b-5p correlates with the higher IL-10 secreted levels by DCs in response to ECOR12 vesicles. This fact, together with the greater induction of miR-146a-5p by ECOR12 MVs, indicates that vesicles from the commensal ECOR12 modulate DCs towards an anti-inflammatory profile.

In addition to miR-146, ECOR12 MVs differentially upregulate other miRNAs, which may account for the attenuated inflammatory response and contribute to the DC’s tolerogenic state, such as members of the miR-125a-99b-let7e cluster. This miR cluster is induced by LPS, TLR2 agonists and pro-inflammatory cytokines by a mechanism that depends on IL-10. As for miR-146b, induction by these mediators displays delayed kinetics as it takes place following IL-10 synthesis. Mature miR125a-5p and let-7e-5p negatively regulate the TLR signaling pathways at different levels by directly targeting the receptor TLR4, downstream signaling proteins and effector cytokines such as IL-12 and TNF-α [54]. Consistently, levels of IL-12 and TNF-α mRNAs were significantly diminished in DCs challenged with ECOR12 MVs compared to EcN-stimulated DCs (see Figure 7). Concerning TLR4 expression, our results showed reduced transcript levels in vesicle-stimulated DCs, independently of the producer strain. In fact, TLR4 has been identified as a direct target of other miRNAs that are also known to be upregulated by EcN MVs (Table 4). MVs from the commensal ECOR12 also differentially induced the expression of other miR-125 family genes, namely miR-125b-5p. This miRNA also destabilizes TNF-α mRNA by binding to its 3′-UTR [55], thus contributing to the attenuation of the inflammatory response. Moreover, ECOR12 MVs also triggered the differential upregulation of miR-24-3p, which has been shown to suppress the secretion of Th1-polarizing cytokines by DCs in response to LPS [70]. Consistently, secreted IL-12 and IFN-γ cytokines and their mRNA levels were significantly reduced in DCs that were exposed to ECOR12 MVs compared to DCs stimulated with MVs from the probiotic EcN. The differential expression of the miR-125 family, miR-let-7e and miR-24, by vesicles from the commensal ECOR12 (upregulation) or the probiotic EcN (non-regulated) is consistent with the ability of induced-DCs to differentially modulate the activation of CD4^+^ T cells towards the Treg or Th1 effector responses [24]. In contrast to the probiotic EcN, MVs from ECOR12 induce a tolerogenic state in DCs and reduce their capacity to drive strong inflammatory Th1 responses against pathogens.

One of the mechanisms that promotes DC-mediated immunosuppression and tolerance is the expression of IDO enzymes that catabolize the amino acid tryptophan. Elevated IDO expression converts mature DCs into tolerogenic antigen-presenting cells that suppress T effector responses by tryptophan depletion and promote regulatory T cells. Human DCs express two IDO isoenzymes, IDO1 and IDO2, which differ in their kinetic parameters and transcriptional regulation. Both genes are activated by LPS, bacterial DNA and IFN-γ, but only IDO2 is constitutively expressed in the absence of stimuli. Recent reports point to IDO2 functional roles in both the tolerogenic capacity of DCs under homeostatic conditions [58] and the modulation of the IDO1-dependent induction of Treg cells under TLR-stimulation conditions [71]. The MIRDB and TargetScan Vent Databases include IDO2 as a potential target of miR-29a-3p, one of the miRNAs that is differentially upregulated by EcN MVs. As expected for the LPS content of bacterial vesicles, both EcN and ECOR12 MVs induced IDO2 expression in mo-DCs, but mRNA levels were significantly lower in EcN-treated cells. In this context, we reported that DCs exposed to EcN MVs induce a lower proportion of CD4^+^CD25^high^ FOXP3 T reg cells than ECOR12 stimulated DCs [24]. Thus, the downregulation of IDO2 by EcN MVs may serve as a mechanism to modulate IDO-dependent tolerogenic responses.

The immunomodulatory properties which were previously described for extracellular vesicles released by the probiotic EcN and the commensal ECOR12 in DCs are in part mediated through the regulation of miRNAs. DCs integrate a diverse array of incoming signals delivered by the MVs released by gut resident microbes and set up specific programs that lead these immune cells to coordinate appropriate T cell responses. The MAMPS included in MVs activate signaling transduction pathways that lead to the activation of transcription factors (NF-κB, AP-1) that trigger the expression of defense and inflammatory mediators. In fact, MVs from *E. coli* microbiota strains elicit similar Th22 (tissue protection and repair) and Th17 (pro-inflammatory, antimicrobial defense) responses. The activation of immune receptors by bacterial vesicles also triggers the upregulation of pro-inflammatory cytokines that direct Th1 responses (protective immunity against pathogens). Consistently, EcN MVs trigger the potent activation of IFN-γ and IL-12 expression, a mechanism that is compatible with the capacity of this probiotic to protect against viral enteric infections. In contrast, DCs exposed to the MVs of the commensal ECOR12 show reduced expression of Th1-driven cytokines but increased expression of anti-inflammatory IL-10. The differential upregulation of specific miRNAs that attenuate the inflammatory response by targeting IL-12, IFN-γ and TNF-α effector cytokines and adaptor proteins downstream TLR pathways may contribute to the anti-inflammatory/tolerogenic action of ECOR12 MVs.

## 5. Conclusions

We are currently living in the microbiome era, and strategies based on the gut microbiota are being explored as potential health promoting therapies or ingredients in functional foods. However, there is still much to learn about the underlying mechanisms of microbiota action. Here, we provide evidence on miRNA regulation by microbiota extracellular vesicles in immune cells. MVs released from gut resident microbes can directly contact DCs lining the intestinal epithelium. By differentially regulating miRNA expression in DCs, microbiota vesicles finely modulate immune training and intestinal homeostasis. Specifically, vesicles from the probiotic EcN prime DCs to mount Th1 responses that are crucial to fight against pathogens and resolve infection, whereas vesicles from the harmless commensal ECOR12 program DCs to orchestrate T regulatory responses that are critical for sustained immune tolerance in the intestine.

## Figures and Tables

**Table 1:** Primers for RT-qPCR analysis of target mRNAs.

Gene ^a^	Sequence	Gene Accession Number
PBX3	F: CAAGTCGGAGCCAATGTG R: ATGTAGCTCAGGGAAAAGTG	NM_001134778.2
MIG6	F: CTACTGGAGCAGTCGCAGTG R: CCTCTTCATGTGGTCCCAAG	AJ276373.1
TNF-α	F: CTTCTGCCTGCTG CACTTTGGA R: TCCCAAAGTAGACCTGCCCAGA	NM_000594.4
TRAF6	F: CCTTTGGCAAATGTCATCTGTG R: CTCTGCATCTTTTCATGGCAAC	NM_145803.3
TLR4	F: ATATTGACAGGAAACCCCATCCA R: AGAGAGATTGAGTAGGGGCATTT	NM_003266.4
IRAK1	F: TCAGAACGGCTTCTACTGCCTG R: TACCCAGAAGGATGTCCAGTCG	NM_001569.4
SOCS1	F: TTGCCTGGAACCATGTGG R: GGTCCTGGCCTCCAGATACAG	NM_003745.1
SHIP1	F: GCTGGAGGAA GAGGACACAG R: AGTCAGCGGGATGTTTCTTG	NM_001017915.3
TAB2	F: CAGCCTGGTCCCTGGACTACT R: ATGAATGGTTGGTGGTTGTGAA	NM_015093.5
IFN-γ	F: TGACCAGAGCATCCAAAAGA R: CTCTTCGACCTCGAAACAGC	NM_000619.3
IDO2	F: AGAAGTGGGCTTTGCTCTGC R: TGGCAAGACCTTACGGACATCTC	NM_002164.6
HPRT-1	F: CCTGGCGTCGTGATTAGTGAT R: AGACGTTCAGTCCTGTCCATAA	NM_000194

^a^ PBX3: pre-B-cell leukemia transcription factor 3, MIG6: mitogen-inducible gene 6, TNF-α: tumor necrosis factor alfa, TRAF6: TNF receptor associated factor 6, TLR4: Toll-like receptor 4, IRAK1: interleukin-1 receptor-associated kinase 1, SOCS1: suppressor of cytokine signaling 1, SHIP1: inositol polyphosphate 5-phosphatase 1, TAB2: TGF-beta activated kinase 1 (MAP3K7) binding protein 2, IFN-γ: interferon gamma, IDO2: indoleamine- pyrrole 2,3-dioxygenase 2, HPRT-1, hypoxanthine phosphoribosyltransferase.

**Table 2:** Statistics of raw, trimmed and mapped sequencing reads.

Group/ Sample	GEO Accession Number	# Reads	# Reads after Trimming	# Uniquely Mapped Reads	% Uniquely Mapped Reads	# Multi- Mapped Reads	% Multi- Mapped Reads
**Control-1**	GSM4286621	43.177.375	39.844.522	33.379.572	83.77	163.725	0.41
**Control-2**	GSM4286622	42.027.544	41.121.701	28.037.324	68.18	323.871	0.79
**Control-3**	GSM4286623	42.035.628	40.198.491	32.221.679	80.16	213.212	0.53
**EcN-MVs-1**	GSM4286624	41.710.678	40.507.274	32.961.507	81.37	181.397	0.45
**EcN-MVs-2**	GSM4286625	37.508.894	34.316.470	26.739.425	77.92	183.188	0.53
**EcN-MVs-3**	GSM4286626	34.990.472	33.402.664	27.717.617	82.98	117.631	0.35
**ECOR12-MVs-1**	GSM4286627	15.384.378	15.125.212	12.702.801	83.98	44.291	0.29
**ECOR12-MVs-2**	GSM4286628	19.955.575	19.682.866	16.032.149	81.45	60.202	0.31
**ECOR12-MVs-3**	GSM4286629	20.245.746	20.008.948	16.915.467	84.54	53.680	0.27

**Table 3:** Expression levels of selected miRNAs in vesicle-stimulated DCs compared to untreated control.

Expression	miRNa	DC + ECOR12 MVs vs. Control			DC + EcN MVs vs. Control		
		Fold Change	log2 Fold Change	Padj	Fold Change	log2 Fold Change	Padj
Common UP EcN ECOR12	hsa-miR-155-5p	10.93	3.45	5.23 × 10^−115^	10.34	3.37	7.1 × 10^−110^
	hsa-let-7i-3p	2.83	1.5	2.84 × 10^−25^	2.97	1.57	1.83 × 10^−28^
	hsa-miR-146a-5p	7.89	2.98	1.89 × 10^−44^	1.79	0.84	3.5 × 10^−4^
UP ECOR12 NOT EcN	hsa-miR-146b-5p	5.86	2.55	3.34 × 10^−30^	0.92	(−0.10)	8.2 × 10^−1^
	hsa-miR-24-3p	5.98	2.58	3.2 × 10^−25^	1.41	0.5	1.25 × 10^−1^
	hsa-miR-125b-5p	3.23	1.69	2.5 × 10^−6^	(−0.23)	0.85	7.3 x 10^−1^
	hsa-miR-125a-5p	1.91	0.93	1.74 × 10^−7^	1.15	0.2	4.67 × 10^−1^
	hsa-miR-99b-5p	2.36	1.24	1.66 × 10^−5^	1.06	0.08	8.99 × 10^−1^
	hsa-let-7e-5p	1.97	0.98	3.33 × 10^−9^	1.23	0.30	1.57 × 10^−1^
	hsa-miR-589-5p	1.82	0.86	3.33 × 10^−9^	−0.62	(−0.69)	3.96 × 10^−6^
UP EcN NOT ECOR12	hsa-miR-33a-3p	−0.54	(−0.95)	1.58 × 10^−6^	1.84	0.88	7.45 × 10^−8^
	hsa-miR-29a-5p	−0.76	(−0.4)	8.49 × 10^−2^	1.54	0.62	1.49 × 10^−3^

**Table 4:** miRNA target genes selected for validation.

Target	miRNA	References
SHIP1	miR-155-5p	[49]
SOCS1	miR-155-5p, let-7i-3p	[50,51]
TAB2	miR-155-5p	[50]
TRAF6	miR-146a-5p, miR-146b-5p	[52]
TLR4	miR-146a-5p, miR125a-5p	[53,54]
IFN-γ	miR-24-3p	MIRDB and TargetScan Vent
TNF-α	miR-125a-5p, let-7e-5p, mir125-5p	[54,55]
IL-12	miR-125a-5p, let-7e-5p	[54]
IDO2	miR-29a-5p	MIRDB and TargetScan Vent
MIG6	miR-589-5p	[56]
PBX3	miR-33a-3p	[57]

## Supplementary Materials

The following are available online at https://www.mdpi.com/2073-4409/9/6/1534/s1, Figure S1: Quantification of DC maturation markers, Figure S2: Heatmap of common miRNAs downregulated by EcN and ECOR12 MVs compared to the control untreated DC group, Figure S3: Heatmaps of miRNA differentially downregulated by EcN MVs (left) or ECOR12 MVs (right) compared to the control untreated DC group, Figure S4: Enriched Molecular Functions and Cellular Components ontologies (GO) of the top 23 upregulated miRNAs targets, Figure S5: Mapping of the 29 enriched Biological Processes (see Figure 10) onto the Gene Ontology tree by the QuickGO tool, Figures S1–S5 legends, Table S1: MiRCURY LNA primers (Qiagen) for RT-qPCR validation of selected miRNAs, Table S2: Differentially expressed miRNAs in response to EcN and/or ECOR12, Table S3: Top 23 miRNAs targets in the indicated databases.
