# Supplementary material for: Transcriptomic microRNA Profiling of Dendritic Cells in Response to Gut Microbiota-Secreted Vesicles
Source: Cells. 2020 Jun 23;9(6):1534. doi: 10.3390/cells9061534 (PMC7349327; doi:10.3390/cells9061534)
Supplement: Supplementary file 1 [file cells-09-01534-s001.zip › SUPPLEMENTAL/Fig S1-S5 Legends.pdf]

Supplementary Figure Legends

Figure S1. **Quantification of DC maturation markers**. Mo-DCs were incubated with MVs (10 µg/ml) from the indicated *E. coli* strains for 24 h or kept in DC medium as a control of immature DCs. Expression of CD209 (DC-SING) and CD83 was analysed by flow cytometry. The graph shows fold-changes in the mean fluorescence intensity (mean ± SEM) of the respective marker expression compared to control immature-DCs (dotted line). Data are from three independent donors performed in triplicate. Statistical differences were evaluated by one-way ANOVA followed by Bonferroni's test. *p <0.05 versus control immature-DCs.

Figure S2. Heatmaps of common miRNA downregulated by both EcN MVs and ECOR12 MVs compared to the control untreated DC group. The color scale next to the panel illustrates the relative expression level of the indicated miRNAs in each sample. Downregulated miRNAs were selected based on FDR-adjusted p-value < 0.005 and log2 Fold Change > 0.6.

Figure S3. Heatmaps of miRNA differentially downregulated by EcN MVs (left) or by ECOR12 MVs (right) compared to the control untreated DC group. The color scale next to each panel illustrates the relative expression level of the indicated miRNAs in each sample. Downregulated miRNAs were selected based on FDR-adjusted p-value < 0.005 and log2 Fold Change > 0.6.

Figure S4. Enriched Molecular Functions and Cellular Components (Gene Ontologies) of the top 23 up-regulated miRNAs targets. Analysis was based on the 23-top upregulated miRNAs by both EcN and ECOR12 MVs when compared to the control untreated DC group. The size of the dots corresponds to the number of genes in the ontology and the colour to the GOstats hypergeometric test P-value. The ontologies are shown only if they were found enriched in at least 3 of the miRNAs target lists.

Figure S5. Mapping of the 29 enriched Biological Processes (see Figure 10) onto the Gene Ontology tree by the QuickGO tool.
