# Supplementary material for: Transcriptomic microRNA Profiling of Dendritic Cells in Response to Gut Microbiota-Secreted Vesicles
Source: Cells. 2020 Jun 23;9(6):1534. doi: 10.3390/cells9061534 (PMC7349327; doi:10.3390/cells9061534)
Supplement: Supplementary file 1 [file cells-09-01534-s001.zip › SUPPLEMENTAL/S3.pdf]

miRNAs differentially Down−regulated only by EcN MVs

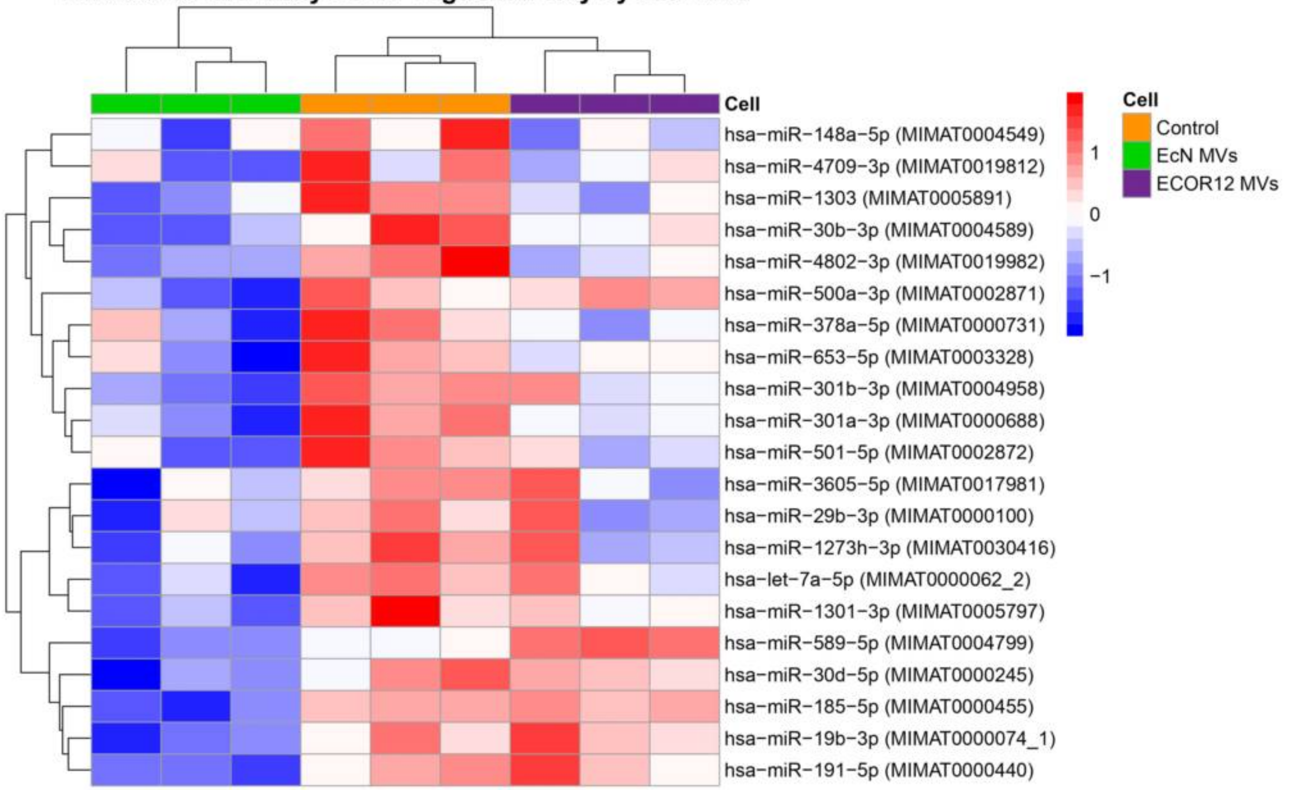

miRNAs differentially Down−regulated only by EcN MVs

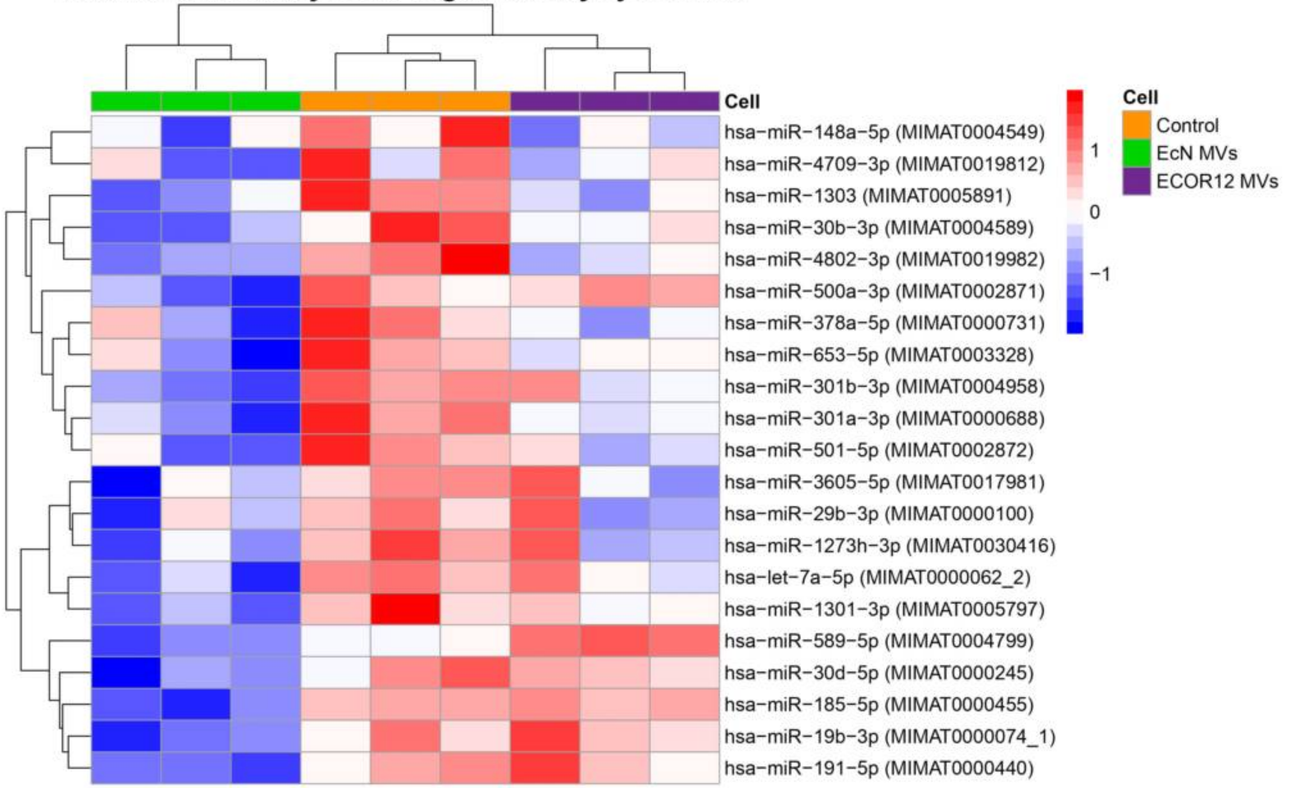
Cell
hsa−miR−148a−5p (MIMAT0004549)
hsa−miR−4709−3p (MIMAT0019812)
hsa−miR−1303 (MIMAT0005891)
hsa−miR−30b−3p (MIMAT0004589)
hsa−miR−4802−3p (MIMAT0019982)
hsa−miR−500a−3p (MIMAT0002871)
hsa−miR−378a−5p (MIMAT0000731)
hsa−miR−653−5p (MIMAT0003328)
hsa−miR−301b−3p (MIMAT0004958)
hsa−miR−301a−3p (MIMAT0000688)
hsa−miR−501−5p (MIMAT0002872)
hsa−miR−3605−5p (MIMAT0017981)
hsa−miR−29b−3p (MIMAT0000100)
hsa−miR−1273h−3p (MIMAT0030416)
hsa−let−7a−5p (MIMAT0000062_2)
hsa−miR−1301−3p (MIMAT0005797)
hsa−miR−589−5p (MIMAT0004799)
hsa−miR−30d−5p (MIMAT0000245)
hsa−miR−185−5p (MIMAT0000455)
hsa−miR−19b−3p (MIMAT0000074_1)
hsa−miR−191−5p (MIMAT0000440)
1
0
−1
Cell
Control
EcN MVs
ECOR12 MVs
