# Supplementary material for: Transcriptomic microRNA Profiling of Dendritic Cells in Response to Gut Microbiota-Secreted Vesicles
Source: Cells. 2020 Jun 23;9(6):1534. doi: 10.3390/cells9061534 (PMC7349327; doi:10.3390/cells9061534)
Supplement: Supplementary file 1 [file cells-09-01534-s001.zip › SUPPLEMENTAL/Table S1.docx]

**Table S1. MiRCURY LNA primers (Qiagen) for RT-qPCR validation of selected miRNAs**

| **Primer ID** | **Accesion number miRbase** | **miRNA** |
| --- | --- | --- |
| hsa-miR-146b-5p | MIMAT0002809 | UGAGAACUGAAUUCCAUAGGCUG |
| hsa-miR-146a-5p | MIMAT0000449 | UGAGAACUGAAUUCCAUGGGUU |
| hsa-miR-24-3p | MIMAT0000080 | UGGCUCAGUUCAGCAGGAACAG |
| hsa-miR-29a-5p | MIMAT0004503 | ACUGAUUUCUUUUGGUGUUCAG |
| hsa-miR-33a-3p | MIMAT0004506 | CAAUGUUUCCACAGUGCAUCAC |
| hsa-miR-589-5p | MIMAT0004799 | UGAGAACCACGUCUGCUCUGAG |
| hsa-miR-155-5p | MIMAT0000646 | UUAAUGCUAAUCGUGAUAGGGGUU |
| hsa-let-7i-3p | MIMAT0004585 | CUGCGCAAGCUACUGCCUUGCU |
| hsa-miR-125b-5p | MIMAT0000423 | UCCCUGAGACCCUAACUUGUGA |
| hsa-miR-125a-5p | MIMAT0000443 | UCCCUGAGACCCUUUAACCUGUGA |
| hsa-miR-99b-5p | MIMAT0000689 | CACCCGUAGAACCGACCUUGCG |
| hsa-let-7e-5p | MIMAT0000066 | UGAGGUAGGAGGUUGUAUAGUU |
| hsa−let−7f−5p | MIMAT0000067 | UGAGGUAGUAGAUUGUAUAGUU |
| hsa−miR−421 | MIMAT0003339 | AUCAACAGACAUUAAUUGGGCGC |
| RNU6-1 | 11278 | CACGAATTTGCGTGTCATCCTT |
