# Supplementary figures and images for: Transcriptomic microRNA Profiling of Dendritic Cells in Response to Gut Microbiota-Secreted Vesicles
Source: Cells. 2020 Jun 23;9(6):1534. doi: 10.3390/cells9061534 (PMC7349327; doi:10.3390/cells9061534)

## Maturation Analysis

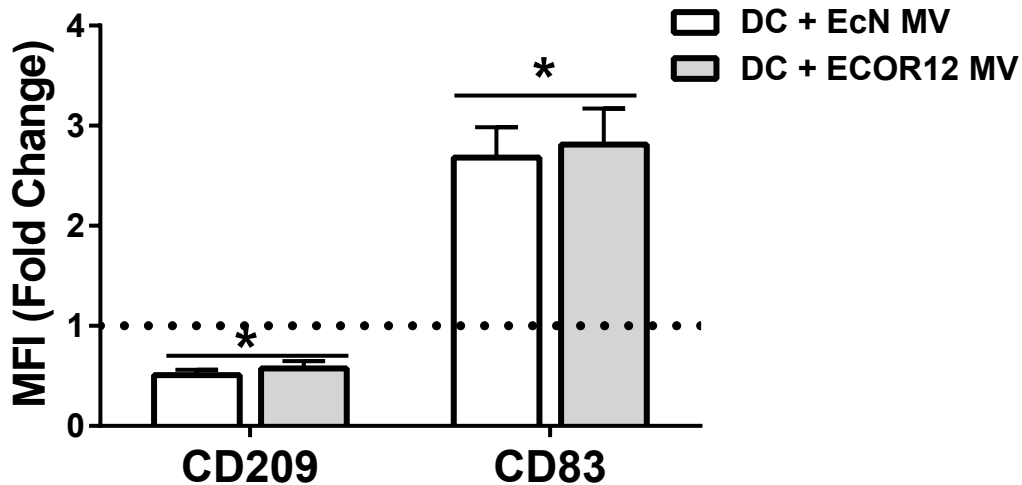

Supplement: Supplementary file 1 [file cells-09-01534-s001.zip › SUPPLEMENTAL/S1.pdf]

# Common Down-regulated miRNAs by EcN MVs and ECOR12 MVs

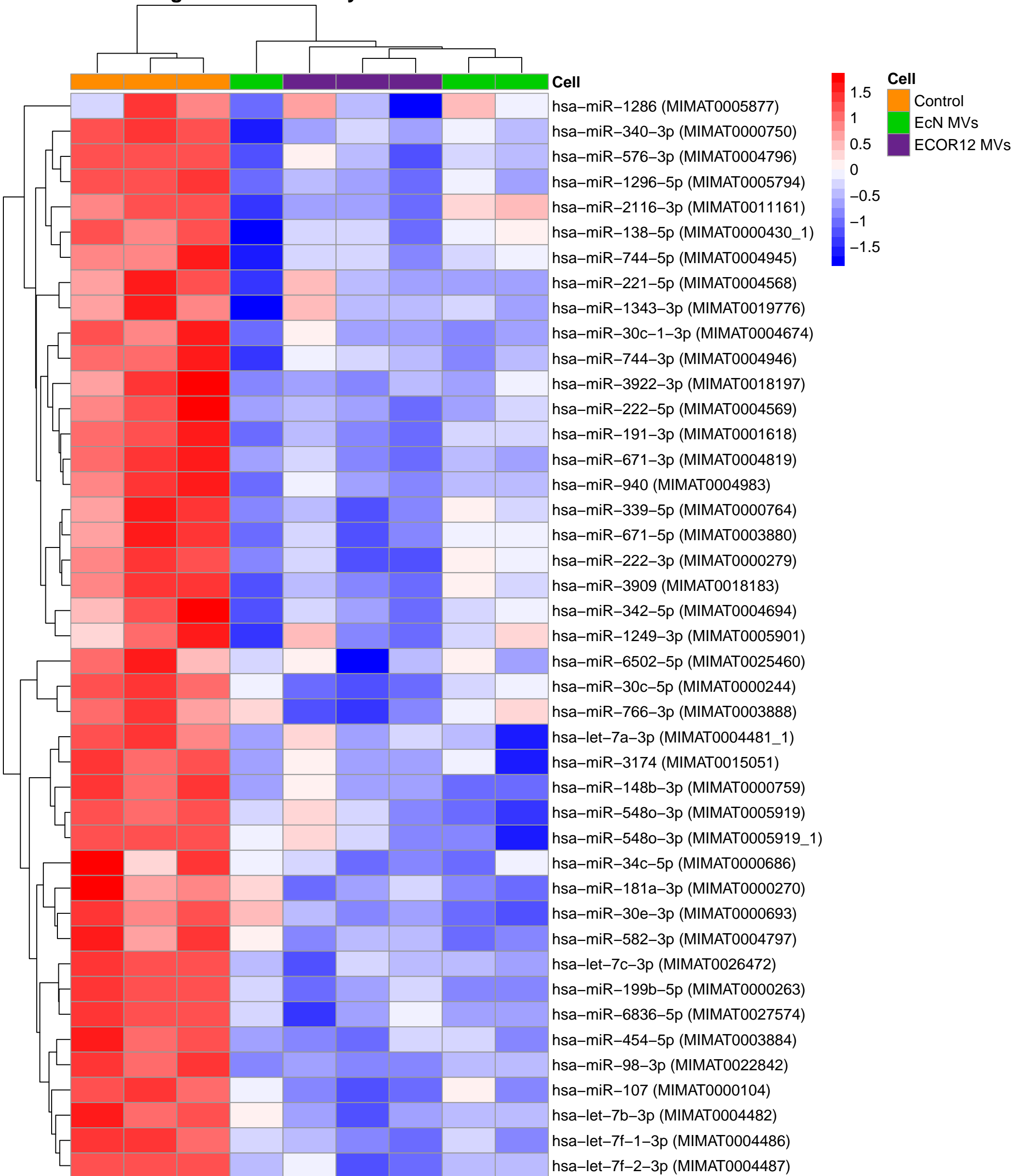

Supplement: Supplementary file 1 [file cells-09-01534-s001.zip › SUPPLEMENTAL/S2.pdf]

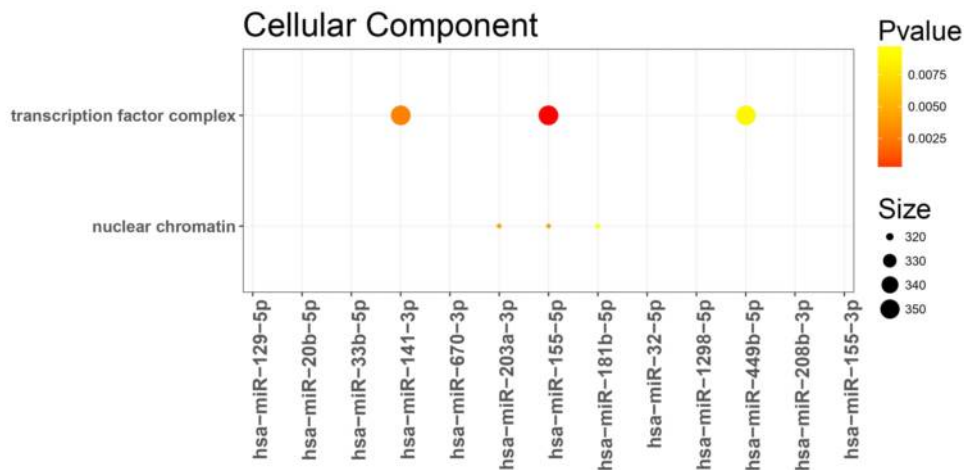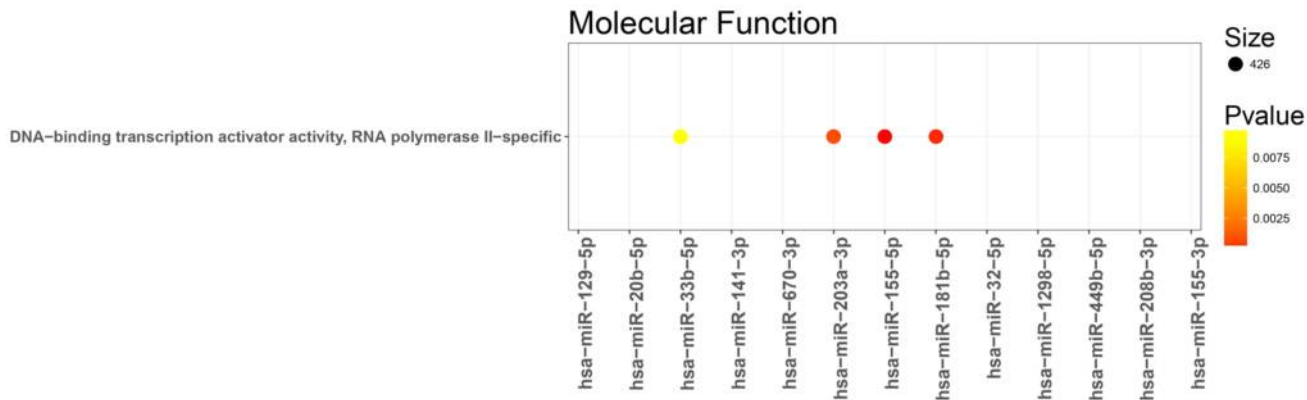

Supplement: Supplementary file 1 [file cells-09-01534-s001.zip › SUPPLEMENTAL/S4.pdf]

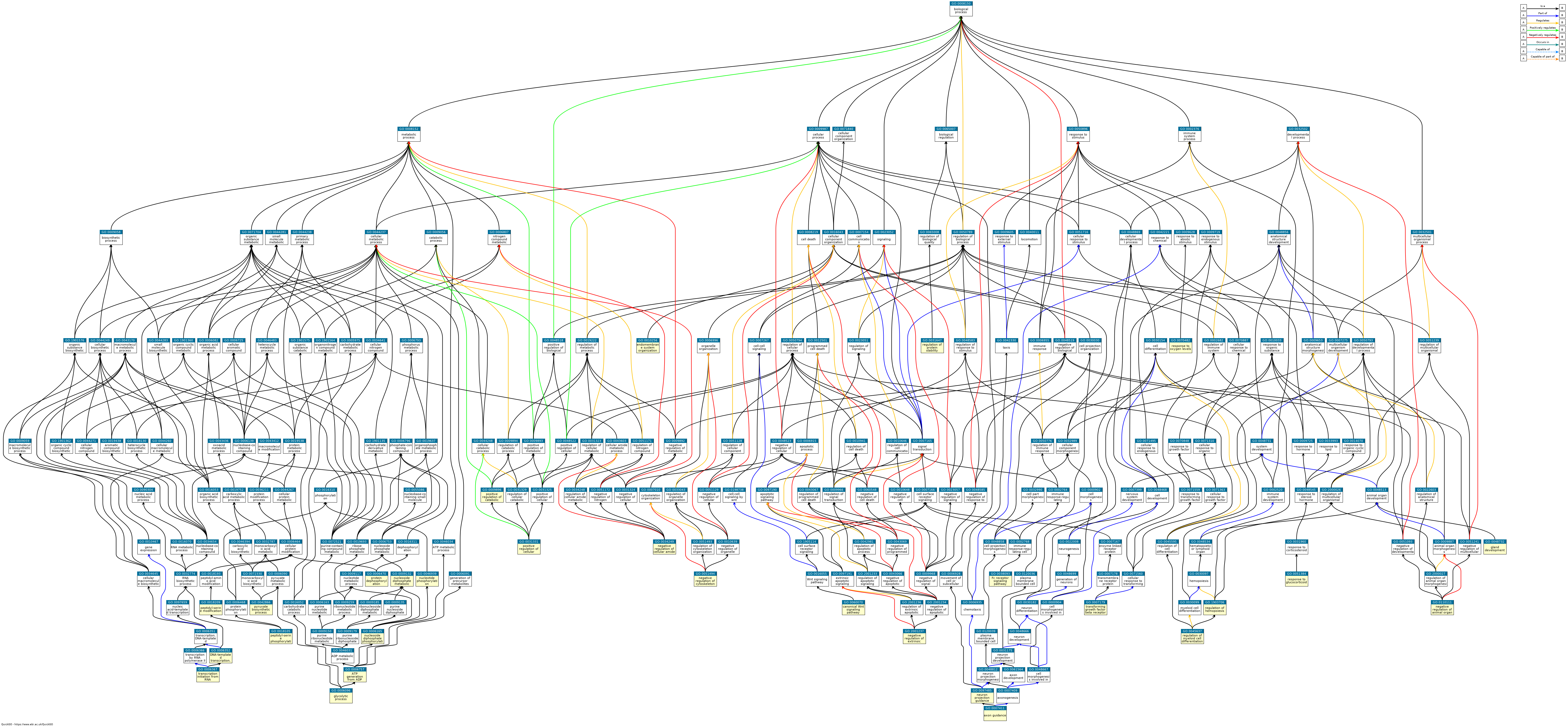

Supplement: Supplementary file 1 [file cells-09-01534-s001.zip › SUPPLEMENTAL/S5.png]
